# Comparison of Efficacies of a Blonanserin Transdermal Patch and Blonanserin Oral Formulation for Psychiatric Symptoms in Patients With Schizophrenia Treated With Laxatives Using a Japanese Claims Database

**DOI:** 10.1002/npr2.70003

**Published:** 2025-02-21

**Authors:** Ken Inada, Hiroyuki Muraoka, Yuji Matsumoto, Daisuke Fukui, Tomomi Watanabe, Yuriko Masuda, Sachie Inoue, Takahiro Masuda

**Affiliations:** ^1^ Department of Psychiatry Kitasato University School of Medicine Sagamihara Kanagawa Japan; ^2^ Medical Science Sumitomo Pharma Co., Ltd. Osaka Japan; ^3^ Global Data Design Office Sumitomo Pharma Co., Ltd. Tokyo Japan; ^4^ CRECON Medical Assessment Inc. Tokyo Japan

**Keywords:** antipsychotics, blonanserin transdermal patch, claims database, laxative, schizophrenia

## Abstract

**Background:**

Laxative use has recently been indicated as a risk factor for hospitalization in patients with schizophrenia. Oral antipsychotic therapy for patients with schizophrenia treated with laxatives may be problematic due to gastrointestinal dysfunction, which affects absorption. Therefore, transdermal patches of antipsychotics may be a suitable alternative. We herein compared the efficacies of a blonanserin (BNS) patch and BNS oral formulation in patients with schizophrenia treated with laxatives.

**Methods:**

A retrospective cohort study was performed using a claims database in Japan provided by DeSC Healthcare Inc. Subjects were BNS patch‐ or BNS oral formulation‐prescribed patients with schizophrenia. The primary outcome was hospitalization to psychiatric wards. The hazard ratio (HR) for hospitalization was estimated using Cox proportional hazards model and adjusted by propensity scores.

**Results:**

Among the 3896 patients identified, 1407 were prescribed laxatives (BNS patch group: *n* = 538, BNS oral group: *n* = 869). Mean ages in the BNS patch and BNS oral groups were 74 and 58 years, respectively. The adjusted HR for hospitalization (BNS patch group vs. BNS oral group) was 1.31 (95% confidence interval; 0.88, 1.94), with no significant difference.

**Conclusions:**

No significant difference was observed in the risk of hospitalization for patients with schizophrenia treated with laxatives between the BNS patch and BNS oral groups. The effectiveness of antipsychotic patches in these patients warrants further research that considers factors such as patch preference in the elderly.

## Abstract 2

Laxative use has recently been identified as a risk factor for hospitalization in patients with schizophrenia, and oral antipsychotic medication may be problematic due to gastrointestinal dysfunction affecting absorption, suggesting that transdermal antipsychotic patches could be a suitable alternative. A retrospective cohort study compared the efficacies of a blonanserin (BNS) patch and BNS oral formulation in patients with schizophrenia treated with laxatives, with the primary outcome being hospitalization to psychiatric wards. The study found no significant difference in the risk of hospitalization between the BNS patch and BNS oral groups, and further research is warranted to consider factors such as patch preference in the elderly.

## Graphical abstract

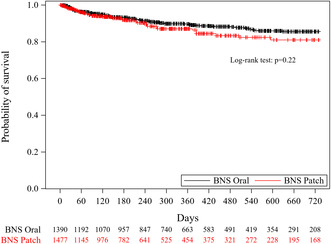

## 1 Introduction

Schizophrenia is a psychiatric disorder that is characterized by various symptoms, such as positive and negative symptoms and cognitive dysfunction. The treatment of schizophrenia is basically medication with antipsychotic drugs, and continuous treatment with antipsychotics is important to reduce the risk of hospitalization to psychiatric wards and death [1, 2].

Patients with schizophrenia often have comorbid physical illnesses, including constipation [3]. Comorbid constipation in schizophrenia may be attributed to various factors, including the administration of medications, such as antipsychotics and anticholinergics, and lifestyle habits, such as diet and exercise. Gastrointestinal diseases with constipation were shown to have an impact on drug absorption by affecting gastrointestinal motor function or permeability [4]. A laxative was found to reduce the absorption of oral antipsychotic drugs in patients with schizophrenia, which may lead to polypharmacy and overdosage with antipsychotics [5]. Furthermore, we recently identified laxative use as a risk factor for hospitalization to psychiatric wards and death in a factor analytical study on schizophrenia using a medical claims database [6]. Therefore, laxative use may be an issue in the antipsychotic medication for the management and treatment of schizophrenia.

Based on these findings, it may be of great clinical significance to identify a highly effective treatment option for psychiatric symptoms in patients with schizophrenia treated with laxatives. In antipsychotic treatment when used in combination with laxatives, it may be appropriate to treat in a manner that is not affected by gastrointestinal dysfunction, such as a reduction in absorption. It is also speculated that the characteristics of transdermal formulations may positively influence the direction of usefulness in the presence of gastrointestinal dysfunction including constipation. Transdermal patches tend to stabilize blood levels through continuous application, which may lead to fewer side effects at the peak and less lack of efficacy at the trough [7]. Fewer side effects would contribute to reduced use of anticholinergics for extrapyramidal symptoms as well as constipation. Maintained efficacy means fewer additional medications that leads to less constipation as a side effect. Furthermore, parenteral administration is favorable for the patients treated with laxatives because it is less stressful on the gastrointestinal tract and has less impact on absorption and metabolism when combined with food or other medications. In this context, antipsychotic patches may be a suitable alternative for patients treated with laxatives in terms of a dosage form because antipsychotics are absorbed transdermally, not through the gastrointestinal tract.

The blonanserin (BNS) transdermal patch (LONASEN Tapes, Sumitomo Pharma Co. Ltd., Japan) is the first antipsychotic patch developed worldwide and is currently the only antipsychotic patch available in Japan. A Phase 3 study on patients with acute schizophrenia demonstrated that the Positive and Negative Syndrome Scale (PANSS) total score at Week 6 was significantly better with 40 and 80 mg BNS patches than with a placebo [8]. A 52‐week administration study on patients with schizophrenia also reported the long‐term safety and efficacy of BNS patches [9].

It remains unclear whether antipsychotic patches are more effective than oral formulations for psychiatric symptoms in patients with schizophrenia treated with laxatives. Therefore, we herein compared the efficacies of a BNS patch and oral formulation in these patients, where hospitalization to psychiatric wards, an indicator of recurrent psychiatric symptoms, was the outcome for efficacy, using a large claims database in Japan.

## 2 Methods

### 2.1 Data Source

A retrospective cohort study was conducted using a claims database provided by DeSC Healthcare Inc. (Tokyo, Japan). The DeSC database consists of anonymized claims data from the National Health Insurance, Employees' Health Insurance, and Advanced Elderly Medical Service System in Japan. The number of individuals enrolled by March 2022 was approximately 7.26 million. Since the DeSC database consists of three types of insurers, the representativeness of the entire population in Japan is considered to be better than that of a database consisting of a single insurer [10]. The BNS transdermal patch was launched in Japan in September 2019; therefore, we used the claims data of patients with schizophrenia (ICD‐10 code: F20) between June 2019 and March 2022.

### 2.2 Patients

The study population was defined as patients meeting the following criteria: (i) receiving a prescription for a BNS patch or oral formulation during or after September 2019; (ii) receiving a diagnosis of schizophrenia (ICD‐10 code: F20) prior to or in the month in which BNS was first prescribed (defined as the index month).

We excluded patients meeting the following criteria: (i) not having a registration period in the database of more than 3 months prior to the index month; (ii) being younger than 15 years old at the index month; (iii) receiving a prescription for a BNS patch or oral formulation within 3 months prior to the index month; (iv) receiving a prescription for both a BNS patch and oral formulation during the index month; (v) being hospitalized to a psychiatric ward within 3 months prior to or in the index month (defined as the look‐back period); (vi) receiving a diagnosis of dementia (ICD‐10 code: F00‐F03) in the look‐back period; (vii) receiving a diagnosis of malignancy (ICD‐10 code: C00‐C97) in the look‐back period; (viii) receiving a prescription for clozapine or electroconvulsive therapy in the look‐back period.

Among eligible patients, we established two cohorts: “patients treated with laxatives” and “patients without laxative use,” depending on the prescription of laxatives in the look‐back period. Patients in each cohort were grouped into the “BNS patch” or “BNS oral” group depending on the prescription on the index date. In a subgroup analysis, we defined “patients treated with laxatives who continued BNS treatment for at least one year” as the population that has not discontinued or switched BNS prescriptions and has not reached the end of the observable period and can be followed for 365 days. Laxatives were defined as drugs classified as WHO‐ATC code: A06A and traditional herbal medicines indicated for constipation in Japan; however, laxatives with indications limited to the pre‐examination preparation were excluded.

### 2.3 Outcome

In the present study, hospitalization was defined as admission to a psychiatric ward. The primary outcome was hospitalization and was evaluated as time‐to‐event data. Patients who switched BNS formulations or received both formulations after the index date were included in the analysis population based on the intent‐to‐treat principle. Patients were followed up from the index date to hospitalization or the following censor, whichever came first: (i) 730 days from the index date; (ii) 15th of the last observable month in the database.

### 2.4 Statistical Analysis

To compare the risk of hospitalization between the BNS patch and oral groups, the crude hazard ratio (HR) and its 95% confidence interval (CI) were estimated by Cox proportional hazards model.

Confounders were adjusted by the inverse probability of treatment weighting method using a propensity score to obtain the adjusted HR. The propensity score (*p*) was estimated by a logistic regression model with patient characteristics as covariables included in Table 1. Weights were calculated as 1/*p* for the BNS patch group and 1/(1‐*p*) for the BNS oral group. Based on a clinical perspective and a previous study [6], the following patient characteristics were included in the model for the propensity score: sex, age (at the index month), number of antipsychotics (at the last prescription in the look‐back period), chlorpromazine equivalent (at the last prescription in the look‐back period), depression (prescribed antidepressants and diagnosed with depression in the look‐back period), prescription of hypnotics (in the look‐back period), and prescription of anticholinergics (in the look‐back period).

**TABLE 1:** Baseline characteristics of patients treated with laxatives.

	Before weighting		After weighting	
	BNS patch	BNS oral	BNS patch	BNS oral
	*n* = 538	*n* = 869	*n* = 1476.80	*n* = 1389.91
Age (years), mean ± SD	73.82 ± 17.23	58.16 ± 18.68	61.96 ± 35.23	63.60 ± 24.03
Sex (male)	226 (42.01%)	268 (30.84%)	462.62 (31.33%)	472.33 (33.98%)
Number of antipsychotics				
0	189 (35.13%)	197 (22.67%)	347.98 (23.56%)	367.90 (26.47%)
1	249 (46.28%)	397 (45.68%)	712.13 (48.22%)	643.09 (46.27%)
2	82 (15.24%)	230 (26.47%)	342.06 (23.16%)	315.43 (22.69%)
≥ 3	18 (3.35%)	45 (5.18%)	74.64 (5.05%)	63.49 (4.57%)
CP equivalent dose (mg), mean ± SD	230.98 ± 368.06	351.53 ± 446.81	321.86 ± 704.60	307.42 ± 537.35
Depression	122 (22.68%)	282 (32.45%)	411.99 (27.90%)	400.02 (28.78%)
Hypnotics	376 (69.89%)	657 (75.60%)	1120.31 (75.86%)	1024.09 (73.68%)
Anticholinergics	89 (16.54%)	275 (31.65%)	428.87 (29.04%)	360.50 (25.94%)
Drug‐free	189 (35.13%)	197 (22.67%)	347.98 (23.56%)	367.90 (26.47%)

*Note:* Data are shown as the number (%) of patients unless otherwise noted.

Abbreviations: BNS, blonanserin; CP, chlorpromazine; SD, standard deviation.

Using the Kaplan–Meier method, adjusted survival curves were generated and differences between the BNS patch and BNS oral groups were tested by the Log‐rank test. Cumulative incidence rates at 3, 6, 12, and 24 months were also estimated.

A subgroup analysis of patients who continued treatment with BNS from the index date for at least 1 year was also performed. *p* < 0.05 were considered to be significant. All statistical analyses were performed with SAS ver. 9.4.

## 3 Results

### 3.1 Patients and Patient Characteristics

**FIGURE 1:**
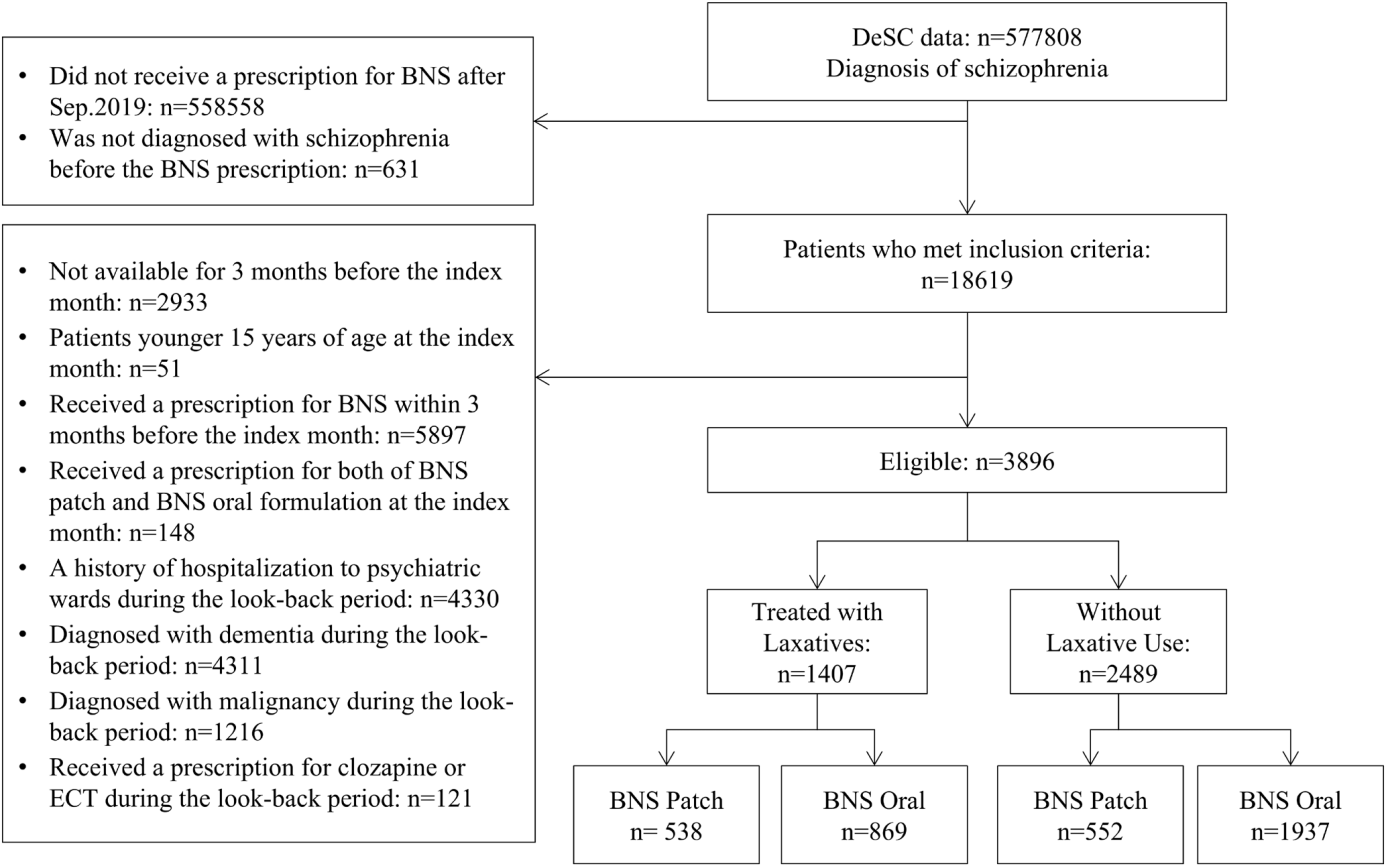
Flowchart of patient selection.

A total of 3896 patients were identified as eligible patients. There were 1407 patients treated with laxatives (BNS patch group: *n* = 538, BNS oral group: *n* = 869) and 2489 patients without laxative use (BNS patch group: *n* = 552, BNS oral group: *n* = 1937) (Figure 1).

**FIGURE 2:**
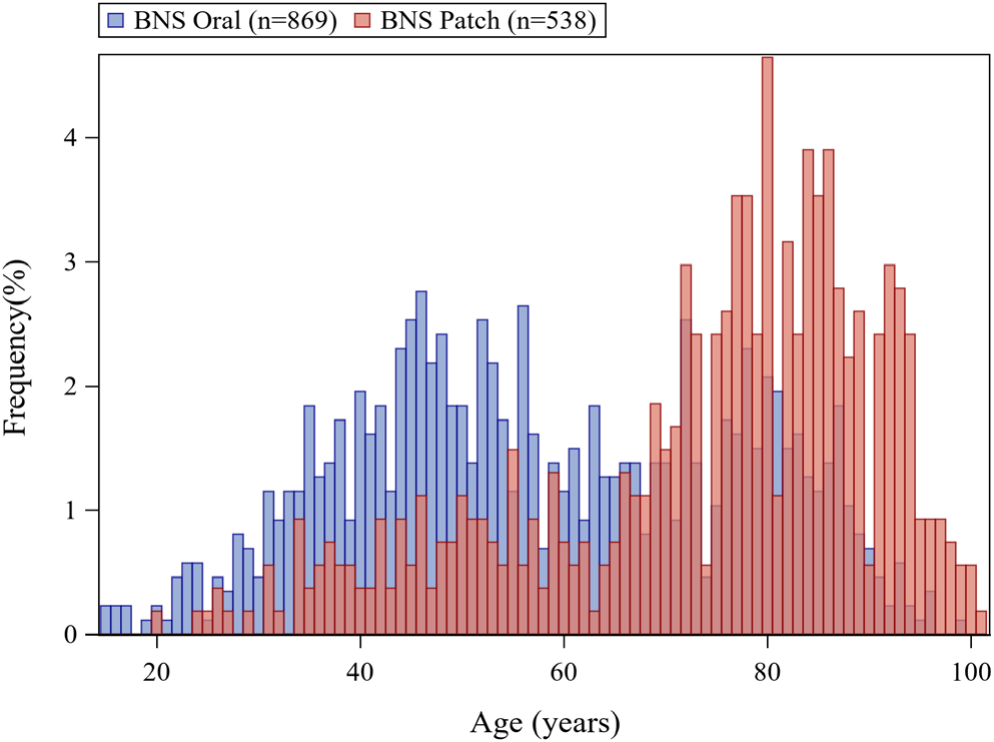
Age distribution of patients treated with laxatives.

The characteristics of patients with schizophrenia treated with laxatives are shown in Table 1. Before weighting, mean age was older in the BNS patch group (73.82 ± 17.23 years, mean ± SD) than that in the BNS oral group (58.16 ± 18.68 years, mean ± SD). The age distribution of patients treated with laxatives by the BNS patch/oral groups (before weighting) is shown in Figure 2. The mean age of patients without laxative use before weighting was also older in the BNS patch group than in the BNS oral group (Table S1). The age distribution of patients without laxative use and all eligible patients by the BNS patch/oral groups (before weighting) are shown in Figure S1.

### 3.2 Analysis Results

#### 3.2.1 Comparisons in Patients Treated With Laxatives

**FIGURE 3:**
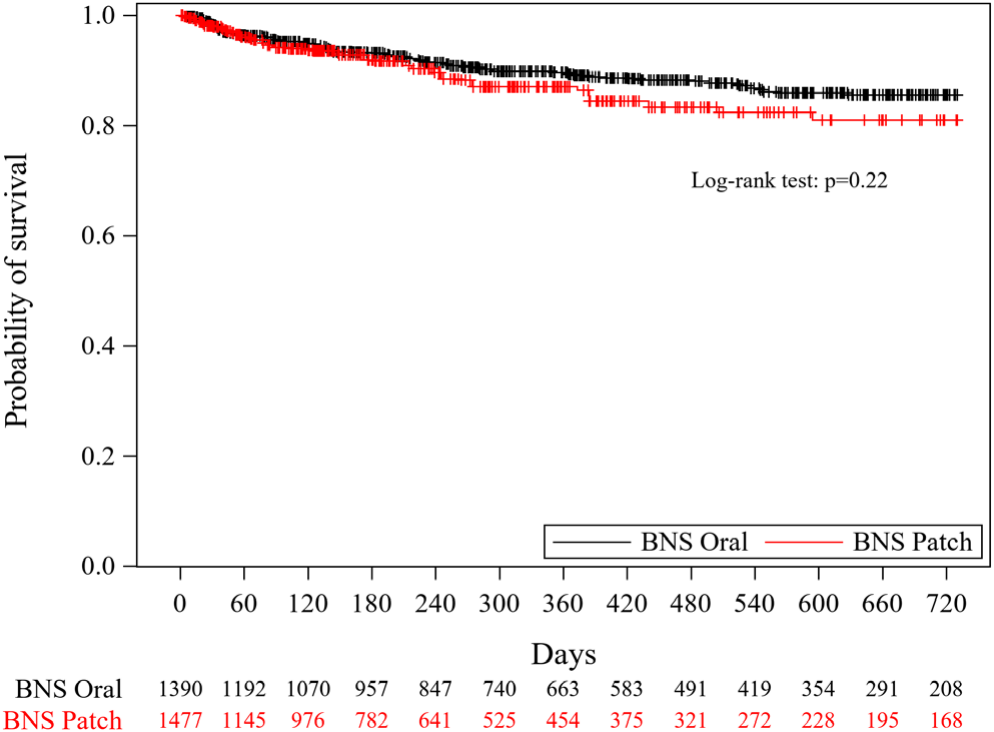
Kaplan–Meier curve of the hospitalization of patients treated with laxatives. The number at the bottom indicates the weighted number of patients at risk.

In patients treated with laxatives, crude HR for hospitalization (BNS patch group vs. BNS oral group) was 1.18 (95% CI; 0.83, 1.68) and adjusted HR was 1.31 (95% CI; 0.88, 1.94), with no significant differences (Figure 3, Table 2). Weighted cumulative incidence rates at 3, 6, 12, and 24 months are shown in Table 2.

**TABLE 2:** Summary of the hospitalization of patients treated with laxatives.

	BNS patch	BNS oral
Weighted cumulative incidence rate		
3 months	5.90%	4.49%
6 months	8.08%	6.84%
12 months	12.97%	10.75%
24 months	19.04%	14.44%
HR, [95% CI]		
Crude	1.18 [0.83, 1.68]	(Reference)
Adjusted	1.31 [0.88, 1.94]	(Reference)

Abbreviations: CI, confidence interval; HR, hazard ratio.

#### 3.2.2 Comparisons in Patients Without Laxative Use

In patients without laxative use, crude HR for hospitalization (BNS patch group vs. BNS oral group) was 1.12 (95% CI; 0.83, 1.52) and adjusted HR was 1.07 (95% CI; 0.78, 1.47), with no significant differences (Figure S2, Table S2). Weighted cumulative incidence rates at 3, 6, 12, and 24 months are shown in Table S2.

#### 3.2.3 Comparisons in Patients Treated With Laxatives Who Continued BNS Treatment for at Least One Year

In patients treated with laxatives who continued BNS treatment for at least 1 year, crude HR for hospitalization (BNS patch group [*n* = 24] vs. BNS oral group [*n* = 140]) was 0.52 (95% CI; 0.07, 3.98) and adjusted HR was 0.27 (95% CI; 0.03, 2.22). The point estimate was lower than 1.00; however, a significant difference was not observed (Figure S3, Table S3).

## 4 Discussion

In the present study, we examined the risk of hospitalization to psychiatric wards in patients with schizophrenia treated with laxatives using a claims database in Japan. No significant differences were observed in the risk of hospitalization between the BNS patch and BNS oral groups.

In our previous study, laxative use was a significant risk factor for hospitalization in patients with schizophrenia who were drug‐free at the time of enrollment and prescribed antipsychotics starting on the index date [6]. We speculated that the decreased gastrointestinal absorption of oral antipsychotics is associated with worsening psychiatric symptoms when using laxatives and, thus, we conducted the present study to investigate whether an antipsychotic patch achieved greater efficacy than oral antipsychotics due to a different absorption pathway in patients with schizophrenia treated with laxatives. However, the risk of hospitalization was not significantly different between the BNS patch and BNS oral groups.

The results obtained may have been affected by the characteristics of the eligible population. The age of patients markedly differed between our previous study and the present study. The mean age of patients in the previous study was 29 years, whereas mean ages in the present study were markedly older, that is, 74 and 58 years in the BNS patch and BNS oral groups, respectively. The elderly are at an increased risk of laxative use [11], and laxative use was suggested as a risk factor for hospitalization in schizophrenia in our previous study. Therefore, an older age may increase the overall risk of hospitalization in the present study population.

Furthermore, baseline antipsychotic treatment differed between our previous study and the present study. At the index date, the eligible population in the previous study comprised antipsychotic‐free subjects, whereas only approximately one‐third to one‐quarter of patients in the present study (i.e., 35% in the BNS patch group and 23% in the BNS oral group) were considered to be antipsychotic‐free. Therefore, the majority of patients in the present study received additional or switched antipsychotic medication at the start of the BNS treatment. The switching of any antipsychotics is associated with an increased risk of hospitalization after discharge within 1 year [12]. Therefore, it is possible that the overall risk of hospitalization in the present study population may have been increased by the inclusion of patients who switched antipsychotic treatment.

Therefore, the hospitalization risk in the study population may have been higher than in our previous study. The increased overall risk of hospitalization in the study population may have led to difficulties investigating the impact of different dosage forms on the risk of hospitalization.

Incidentally, the increased risk of hospitalization due to antipsychotic switching was found to be slightly higher in the first 90 days and subsequently decreased during the one‐year observation period [12]. These findings suggest that the population of patients who maintained the same antipsychotic treatment for more than 1 year may be affected less by antipsychotic switching. From this perspective, in the subgroup analysis of patients who continued BNS treatment for at least 1 year, the risk of hospitalization was lower in the BNS patch group than in the BNS oral group, although not significantly. Furthermore, since atypical antipsychotics cause constipation as a common side effect, management of constipation with laxatives is a more important issue in the long‐term antipsychotic treatment. Thus, in such a long‐term situation, antipsychotic patches may provide a greater benefit to patients treated with laxatives.

Although limited information is currently available on BNS patches, we obtained novel clinical insights into their actual use in the present study. One insight is that the patch formulation may be preferred by the elderly. In patients treated with laxatives, the mean age of patients was older in the BNS patch group than in the BNS oral group (BNS patch group: 74 years, BNS oral group: 58 years). Regarding the preference for patch formulations among the elderly, a previous study on different diseases reported that non‐steroidal anti‐inflammatory patches were used more frequently than oral formulations in elderly patients with osteoarthritis and chronic low back pain [13]. Therefore, the patch formulation may be preferred by the elderly regardless of the disease. Another insight is that the patch formulation may be preferred by patients with comorbid physical conditions, such as gastrointestinal disorders. In this regard, the BNS patch was preferred by patients treated with laxatives. In the present study, the percentage of BNS patches prescribed was higher in patients treated with laxatives than in those without laxative use (treated with laxatives: 538/1407 = 38.2%; without laxative use: 552/2489 = 22.2%). Other studies also showed the benefits of patches. In patients with Alzheimer's disease, the rivastigmine patch is often used because of the advantages of the dosage form, including its use by patients with dysphagia and the avoidance of gastrointestinal dysfunction [14]. These preferences for patches among the elderly and patients with comorbid physical disorders may apply to patients with schizophrenia. The potential effectiveness of the patch may lie behind these preferences. However, given the preference of the elderly for patch formulations and the fact that age is also associated with the occurrence of physical disease, it is likely that age is a confounding factor in the latter insight, and this should be taken into careful consideration.

The present study has several limitations, including those arising from the availability and characteristics of the medical database, which may have affected the results of the analysis. The severity of the digestive symptoms for which laxatives were used was not assessed in the present study and may have affected the outcome; however, it was difficult to define its severity based on claims data. Furthermore, although the dose of antipsychotic drugs may have affected the results obtained, there were no data on the daily doses of topical agents, including the BNS patch, in the claims database. In addition, there were several unmeasured confounders in the present study because they were not included in the database. Another limitation is that schizophrenia was the target disease with established selection criteria in the present study; however, patients with other psychiatric diseases may have been included due to diagnostic misclassifications in the claims database. Moreover, a prospective study would have accurately demonstrated the hypothesis of the present study.

## 5 Conclusions

In the present study, no significant differences were observed in the risk of hospitalization to psychiatric wards for patients with schizophrenia treated with laxatives between the BNS patch and BNS oral groups. We were unable to clarify the clinical question on the appropriate dosage form of antipsychotics for the treatment of psychiatric symptoms in these patients, and the effectiveness of the patch formulation in these patients remains an issue to be investigated. Furthermore, the preference of antipsychotic patches in the elderly and patients treated with laxatives observed in the present study may provide meaningful insights into the actual use of antipsychotic patches in clinical practice.

## Author Contributions

All authors contributed to the study design and interpretation of the results. All authors critically revised the manuscript and approved the final draft.

## Ethics Statement

No ethics review was necessary for this study because it was a secondary analysis of an anonymous patient database.

## Consent

Patient consent was not obtained for this study because it was a secondary analysis of an anonymous patient database.

## Conflicts of Interest

Ken Inada received personal fees from Daiichi‐Sankyo, Eisai, Eli Lilly, Janssen, Lundbeck Japan, Meiji Seika Pharma, Mitsubishi Tanabe Pharma, Mochida, MSD, Nipro, Novartis, Otsuka, Pfizer, Shionogi, Sumitomo Pharma, Yoshitomiyakuhin, and Viatris, and research grant support from Mochida and Sumitomo Pharma. Hiroyuki Muraoka received personal fees from Kyowa, Lundbeck Japan, Janssen, Takeda, Eisai, Meiji‐Seika Pharma, MSD, Otsuka, Sumitomo Pharma, and Viatris. Takahiro Masuda, Yuji Matsumoto, Daisuke Fukui, and Tomomi Watanabe are full‐time employees of Sumitomo Pharma Co. Ltd. Sachie Inoue and Yuriko Masuda are full‐time employees of CRECON Medical Assessment Inc. CRECON Medical Assessment Inc. was paid by Sumitomo Pharma Co. Ltd. to conduct analyses for the study.

## Supporting information


Figure S1.

## Data Availability

Data that support the present results are available from DeSC Healthcare Inc.; however, restrictions apply to the availability of these data, which were used under license for the present study and, thus, are not publicly available. However, data are available from the authors upon reasonable request and with the permission of DeSC Healthcare Inc.
