# Supplementary material for: Comparison of Efficacies of a Blonanserin Transdermal Patch and Blonanserin Oral Formulation for Psychiatric Symptoms in Patients With Schizophrenia Treated With Laxatives Using a Japanese Claims Database
Source: Neuropsychopharmacol Rep. 2025 Feb 21;45(1):e70003. doi: 10.1002/npr2.70003 (PMC11843157; doi:10.1002/npr2.70003)
Supplement: Supplementary file 1 — Figure S1. [file NPR2-45-e70003-s001.docx]

## Table S1 Baseline characteristics of patients without laxative use

|  | Before Weighting | | After Weighting | |
| --- | --- | --- | --- | --- |
|  | BNS Patch | BNS Oral | BNS Patch | BNS Oral |
|  | n=552 | n=1937 | n=2521.53 | n=2485.25 |
| Age (years), mean±SD | 55.26±21.57 | 47.67±18.02 | 48.52±43.66 | 49.22±20.96 |
| Sex (Male) | 232 (42.03%) | 811 (41.87%) | 1,038.34 (41.18%) | 1,038.31 (41.78%) |
| Number of antipsychotics |  |  |  |  |
| 0 | 182 (32.97%) | 583 (30.10%) | 740.33 (29.36%) | 760.22 (30.59%) |
| 1 | 260 (47.10%) | 902 (46.57%) | 1,198.58 (47.53%) | 1,162.45 (46.77%) |
| 2 | 87 (15.76%) | 383 (19.77%) | 480.61 (19.06%) | 469.59 (18.90%) |
| ≥3 | 23 (4.17%) | 69 (3.56%) | 102.02 (4.05%) | 92.98 (3.74%) |
| CP equivalent dose (mg)), mean±SD | 265.36±357.16 | 285.73±398.42 | 293.65±776.62 | 282.53±452.43 |
| Depression | 97 (17.57%) | 506 (26.12%) | 617.37 (24.48%) | 603.84 (24.30%) |
| Hypnotics | 273 (49.46%) | 1,146 (59.16%) | 1,464.18 (58.07%) | 1,419.69 (57.12%) |
| Anticholinergics | 98 (17.75%) | 513 (26.48%) | 653.19 (25.90%) | 612.80 (24.66%) |
| Drug-free | 182 (32.97%) | 583 (30.10%) | 740.33 (29.36%) | 760.22 (30.59%) |

Data are shown as the number (%) of patients unless otherwise noted.

BNS, blonanserin; CP, chlorpromazine; SD, standard deviation

## Table S2. Summary of the hospitalization of patients without laxative use

|  | BNS Patch | BNS Oral |
| --- | --- | --- |
| Weighted cumulative incidence rate |  |  |
| 3 months | 5.17% | 4.90% |
| 6 months | 7.66% | 7.22% |
| 12 months | 11.51% | 10.62% |
| 24 months | 15.49% | 15.50% |
| HR, [95%CI] |  |  |
| Crude | 1.12 [0.83, 1.52] | (reference) |
| Adjusted | 1.07 [0.78, 1.47] | (reference) |

CI, confidence interval; HR, hazard ratio

## Table S3. Summary of the hospitalization of patients treated with laxatives who continued treatment for one year

|  | BNS Patch | BNS Oral |
| --- | --- | --- |
| Weighted cumulative incidence rate |  |  |
| 3 months | 2.21% | 1.93% |
| 6 months | 2.21% | 2.54% |
| 12 months | 2.21% | 4.96% |
| 24 months | 2.21% | 9.46% |
| HR, [95%CI] |  |  |
| Crude | 0.52 [0.07, 3.98] | (reference) |
| Adjusted | 0.27 [0.03, 2.22] | (reference) |

CI, confidence interval; HR, hazard ratio

## Figure S1 Age distribution of the study population

1.
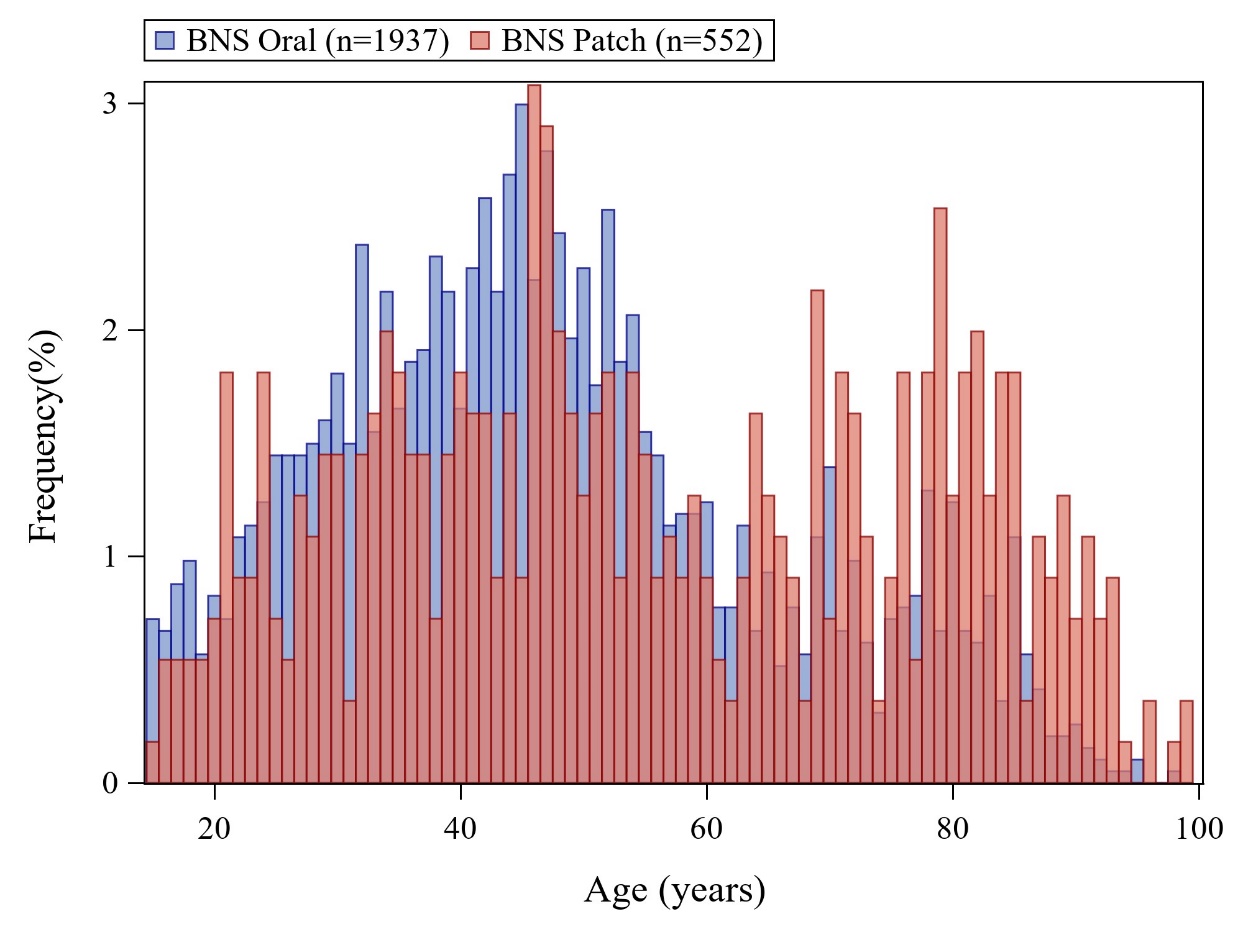
Patients without laxative use
2. Eligible patients


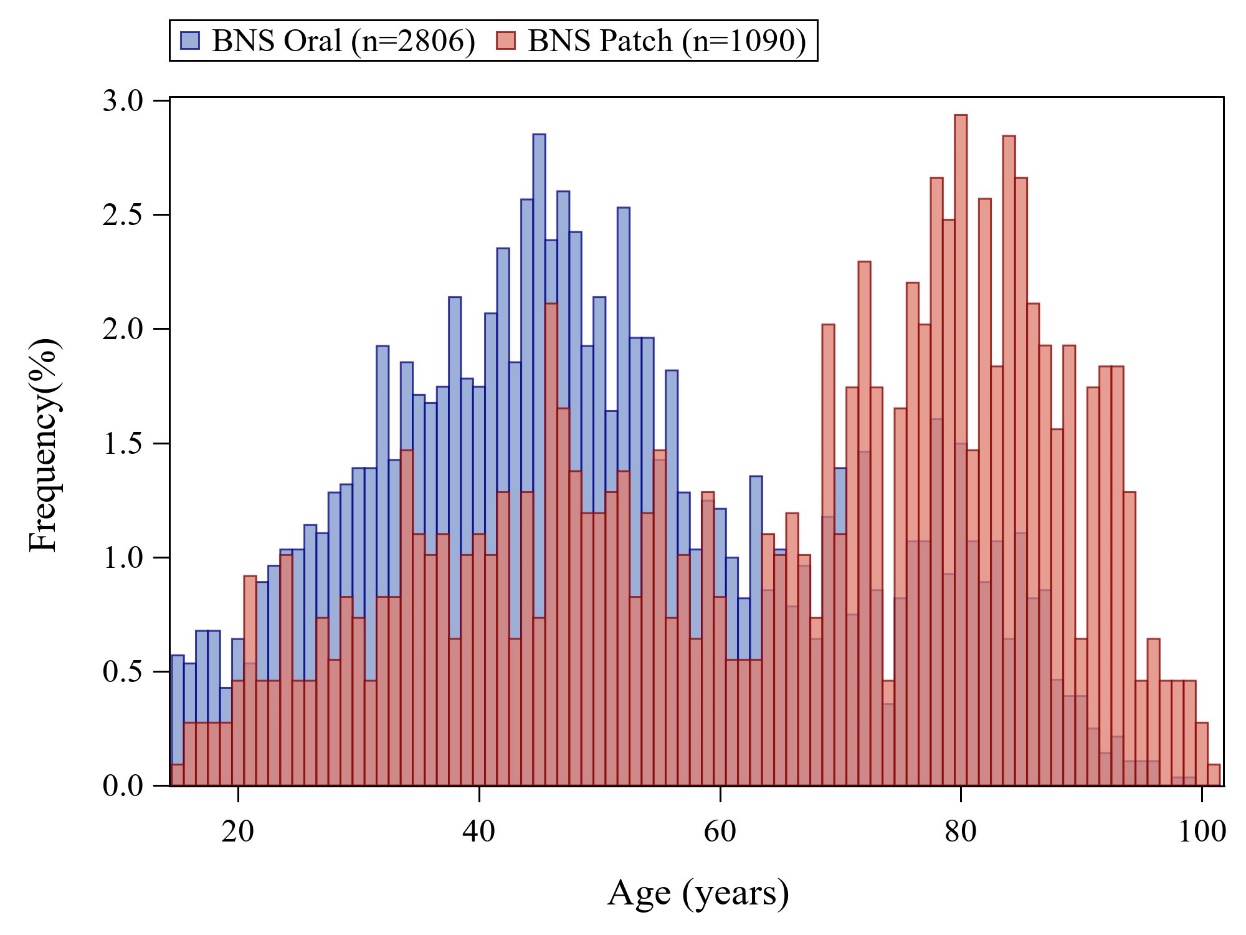


##
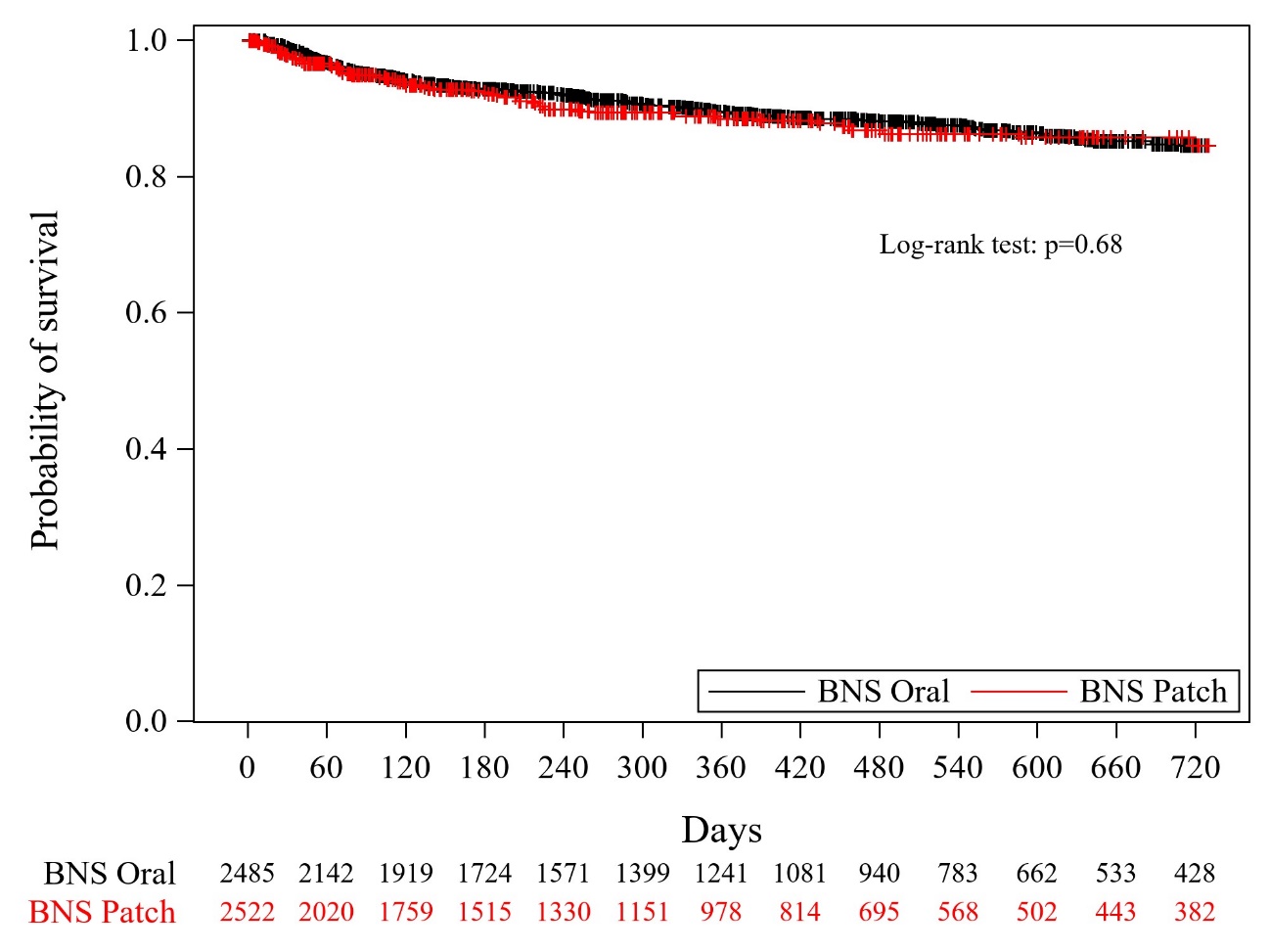
Figure S2. Kaplan-Meier curve of the hospitalization of patients without laxative use

The number at the bottom indicates the weighted number of patients at risk.

##
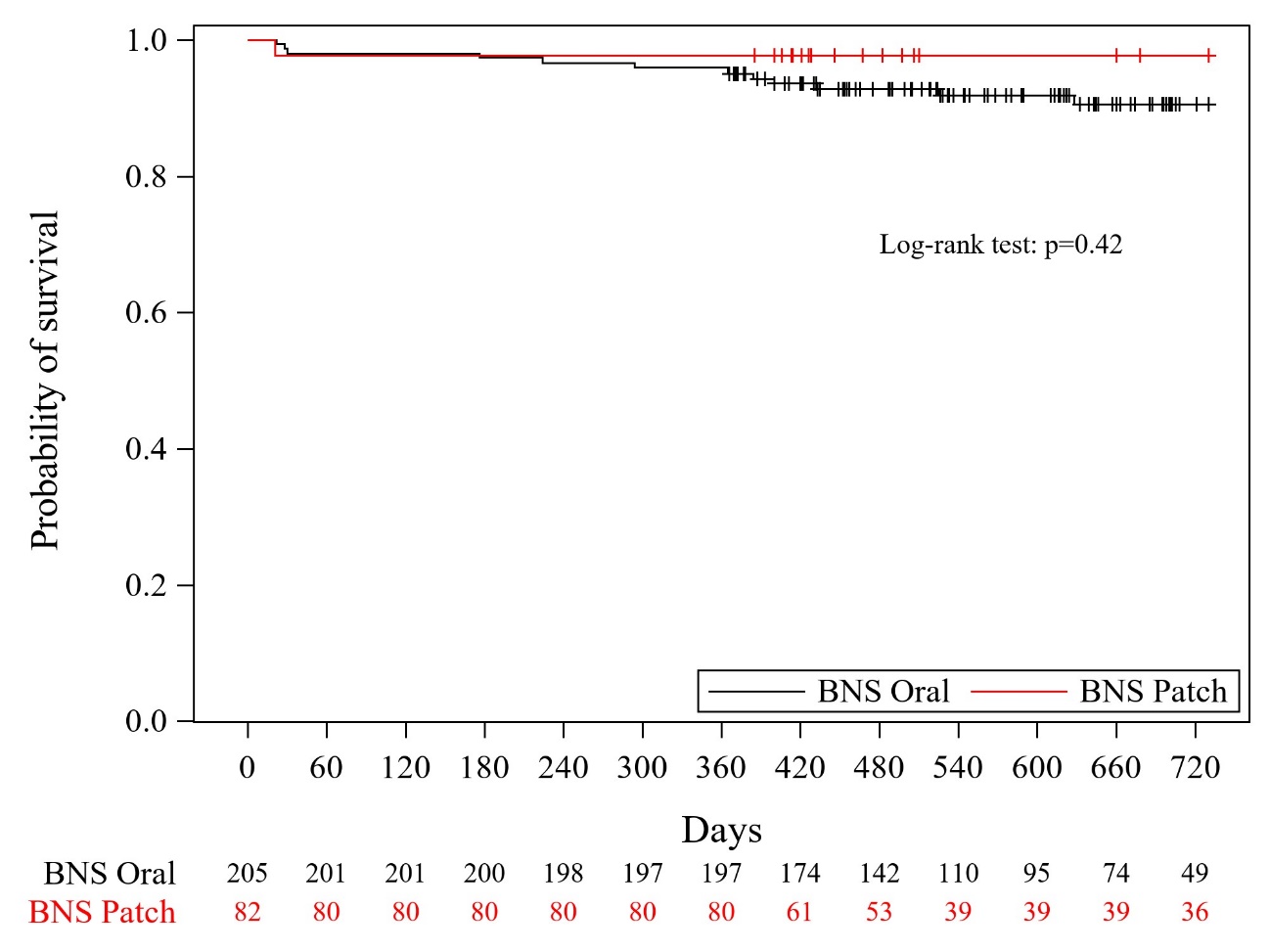
Figure S3. Kaplan-Meier curve of the hospitalization of patients treated with laxatives who continued treatment for one year

The number at the bottom indicates the weighted number of patients at risk.
